# {μ-6,6′-Dimeth­oxy-2,2′-[propane-1,3-diylbis(nitrilo­methyl­idyne)]diphenolato}dimethano­ltrinitratonickel(II)lanthanum(III) methanol disolvate

**DOI:** 10.1107/S1600536808026986

**Published:** 2008-08-30

**Authors:** Fei Liu

**Affiliations:** a College of Chemical Engineering and Materials, Eastern Liaoning University, 325 Wenhua Road, Yuanbao District, Dandong City, Liaoning Province, 118003, People’s Republic of China

## Abstract

In the title dinuclear complex, [NiLa(C_19_H_20_N_2_O_4_)(NO_3_)_3_(CH_3_OH)_2_]·2CH_3_OH, the Ni^II^ ion is coordinated by two O atoms and two N atoms of a Schiff base ligand and by two O atoms of two methanol ligands, forming a slightly distorted octa­hedral geometry. The La^III^ ion is coordinated by six O atoms from three chelating nitrate ligands and four O atoms from the Schiff base ligand, forming a distorted bicapped square-anti­prismatic environment. In the crystal structure, inter­molecular O—H—O hydrogen bonds connect complex mol­ecules and methanol solvent mol­ecules, forming a two-dimensional network.

## Related literature

For the isostructural Pr(III) complex, see: Liu & Zhang (2008 ▶). For a related Sm(III) complex, see: Wang *et al.* (2008 ▶).
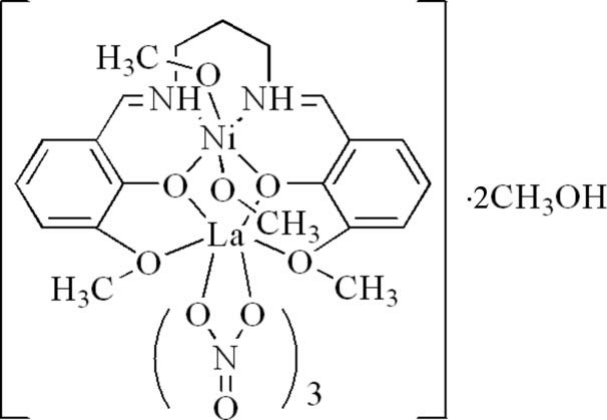


## Experimental

### 

#### Crystal data

[NiLa(C_19_H_20_N_2_O_4_)(NO_3_)_3_(CH_3_OH)_2_]·2CH_3_OH
*M* *_r_* = 852.19
Monoclinic, 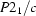

*a* = 13.123 (4) Å
*b* = 11.141 (3) Å
*c* = 22.245 (8) Å
β = 90.911 (13)°
*V* = 3252.0 (17) Å^3^
*Z* = 4
Mo *K*α radiation
μ = 1.96 mm^−1^
*T* = 291 (2) K
0.30 × 0.27 × 0.25 mm

#### Data collection

Rigaku R-AXIS RAPID diffractometer
Absorption correction: multi-scan (*ABSCOR*; Higashi, 1995 ▶) *T*_min_ = 0.594, *T*_max_ = 0.635
29897 measured reflections
7431 independent reflections
6035 reflections with *I* > 2σ(*I*)
*R*_int_ = 0.039

#### Refinement

*R*[*F* ^2^ > 2σ(*F* ^2^)] = 0.032
*wR*(*F* ^2^) = 0.077
*S* = 1.06
7431 reflections
430 parameters
19 restraints
H-atom parameters constrained
Δρ_max_ = 0.67 e Å^−3^
Δρ_min_ = −0.56 e Å^−3^

### 

Data collection: *RAPID-AUTO* (Rigaku, 1998 ▶); cell refinement: *RAPID-AUTO*; data reduction: *CrystalStructure* (Rigaku/MSC, 2002 ▶); program(s) used to solve structure: *SHELXS97* (Sheldrick, 2008 ▶); program(s) used to refine structure: *SHELXL97* (Sheldrick, 2008 ▶); molecular graphics: *SHELXTL* (Sheldrick, 2008 ▶); software used to prepare material for publication: *SHELXL97*.

## Supplementary Material

Crystal structure: contains datablocks I. DOI: 10.1107/S1600536808026986/lh2680sup1.cif

Structure factors: contains datablocks I. DOI: 10.1107/S1600536808026986/lh2680Isup2.hkl

Additional supplementary materials:  crystallographic information; 3D view; checkCIF report

## Figures and Tables

**Table 1:** Hydrogen-bond geometry (Å, °)

*D*—H⋯*A*	*D*—H	H⋯*A*	*D*⋯*A*	*D*—H⋯*A*
O17—H33⋯O16	0.85	2.10	2.665 (7)	124
O15—H25⋯O16^i^	0.85	1.83	2.681 (5)	174
O14—H21⋯O12^ii^	0.85	2.34	3.169 (5)	165
O16—H29⋯O15^iii^	0.85	2.35	2.681 (5)	104

Symmetry codes: (i) 
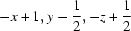
; (ii) 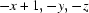; (iii) 
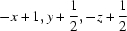
.

**Fig. 1.**
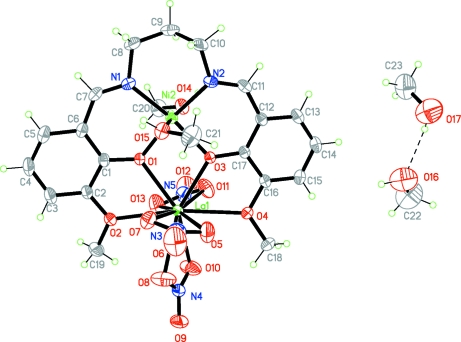
The molecular structure of (I), showing 40% probability displacement ellipsoids. The dashed line indicates a hydrogen bond.

## supplementary crystallographic information

### Comment

As shown in Fig. 1, the hexadentate Schiff base ligand links Ni and La atoms into a dinuclear complex through two phenolate O atoms, which is the same as the bonding in the isostructural Pr(III) complex of the same ligand (Liu & Zhang, 2008) and a related Cu(II)/Sm(III) complex (Wang *et al.*, 2008). The La^III^ ion in (I) is ten-coordinated by four oxygen atoms from the ligand and six oxygen atoms from three nitrate ions. The Ni^II^ center is six-coordinate by two nitrogen atoms and two oxygen atoms from the ligand and two methanol oxygen atoms. The are two solvent methanol molecules for each complex molecule. In the crystal structure, intermolecular O—H—O hydrogen bonds connect complex molecules and methanol solvent molecules to form two-dimension structure.

### Experimental

The title complex was obtained by the treatment of nickel(II) acetate tetrahydrate (0.0622 g, 0.25 mmol) with the Schiff base (0.0855 g, 0.25 mmol) in methanol (25 ml) at room temperature. Then the mixture was refluxed for 3 h after the addition of lanthanum (III) nitrate hexahydrate (0.1082 g, 0.25 mmol). The reaction mixture was cooled and filtered; diethyl ether was allowed to diffuse slowly into the solution of the filtrate. Blue single crystals were obtained after several days. Analysis calculated for C_23_H_36_NiN_5_O_17_La: C, 32.48; H, 4.02; N, 8.27; found: C, 32.49; H, 4.03; N, 8.24

### Refinement

H atoms bound to C atoms were placed in calculated positions and treated as riding on their parent atoms, with C—H = 0.93 Å (aromatic C), C—H = 0.97 Å (methylene C) and with *U*_iso_(H) = 1.2Ueq(C) or C—H = 0.96 Å (methly C) and with *U*_iso_(H) = 1.5Ueq(C). H atoms bond to O atoms of methanol were initially located in a difference Fourier map, but were subsequently treated as riding on their parent atoms, with O—H = 0.85 Å, and with *U*_iso_(H) = 1.2U_eq_(O).

### Crystal data

**Table:**

[NiLa(C_19_H_20_N_2_O_4_)(NO_3_)_3_(CH_3_OH)_2_]·2CH_3_OH	*F*_000_ = 1716
*M**_r_* = 852.19	*D*_x_ = 1.739 Mg m^−^^3^
Monoclinic, *P*2_1_/*c*	Mo *K*α radiation λ = 0.71073 Å
Hall symbol: -P 2ybc	Cell parameters from 24213 reflections
*a* = 13.123 (4) Å	θ = 6.0–55.0º
*b* = 11.141 (3) Å	µ = 1.96 mm^−^^1^
*c* = 22.245 (8) Å	*T* = 291 (2) K
β = 90.911 (13)º	Block, green
*V* = 3252.0 (17) Å^3^	0.30 × 0.27 × 0.25 mm
*Z* = 4	

### Data collection

**Table:**

Rigaku R-AXIS RAPID diffractometer	7431 independent reflections
Radiation source: fine-focus sealed tube	6035 reflections with *I* > 2σ(*I*)
Monochromator: graphite	*R*_int_ = 0.039
*T* = 291(2) K	θ_max_ = 27.5º
ω scans	θ_min_ = 3.0º
Absorption correction: multi-scan(ABSCOR; Higashi, 1995)	*h* = −16→17
*T*_min_ = 0.594, *T*_max_ = 0.635	*k* = −14→14
29897 measured reflections	*l* = −28→28

### Refinement

**Table:**

Refinement on *F*^2^	Secondary atom site location: difference Fourier map
Least-squares matrix: full	Hydrogen site location: inferred from neighbouring sites
*R*[*F*^2^ > 2σ(*F*^2^)] = 0.032	H-atom parameters constrained
*wR*(*F*^2^) = 0.078	*w* = 1/[σ^2^(*F*_o_^2^) + (0.0311*P*)^2^ + 2.8126*P*] where *P* = (*F*_o_^2^ + 2*F*_c_^2^)/3
*S* = 1.07	(Δ/σ)_max_ = 0.005
7431 reflections	Δρ_max_ = 0.67 e Å^−^^3^
430 parameters	Δρ_min_ = −0.56 e Å^−^^3^
19 restraints	Extinction correction: none
Primary atom site location: structure-invariant direct methods	

### Special details

**Table:**

Geometry. All e.s.d.'s (except the e.s.d. in the dihedral angle between two l.s. planes) are estimated using the full covariance matrix. The cell e.s.d.'s are taken into account individually in the estimation of e.s.d.'s in distances, angles and torsion angles; correlations between e.s.d.'s in cell parameters are only used when they are defined by crystal symmetry. An approximate (isotropic) treatment of cell e.s.d.'s is used for estimating e.s.d.'s involving l.s. planes.
Refinement. Refinement of *F*^2^ against ALL reflections. The weighted *R*-factor *wR* and goodness of fit *S* are based on *F*^2^, conventional *R*-factors *R* are based on *F*, with *F* set to zero for negative *F*^2^. The threshold expression of *F*^2^ > σ(*F*^2^) is used only for calculating *R*-factors(gt) *etc*. and is not relevant to the choice of reflections for refinement. *R*-factors based on *F*^2^ are statistically about twice as large as those based on *F*, and *R*- factors based on ALL data will be even larger.

### Fractional atomic coordinates and isotropic or equivalent isotropic displacement parameters (Å^2^)

**Table:**

	*x*	*y*	*z*	*U*_iso_*/*U*_eq_	
C1	0.8780 (2)	0.0634 (3)	0.09172 (14)	0.0366 (7)	
C2	0.9465 (2)	0.1419 (3)	0.06390 (15)	0.0401 (7)	
C3	1.0476 (3)	0.1149 (4)	0.05748 (18)	0.0537 (9)	
H1	1.0910	0.1693	0.0391	0.064*	
C4	1.0845 (3)	0.0066 (4)	0.0784 (2)	0.0643 (12)	
H2	1.1531	−0.0123	0.0743	0.077*	
C5	1.0206 (3)	−0.0725 (4)	0.10520 (19)	0.0589 (10)	
H3	1.0465	−0.1451	0.1193	0.071*	
C6	0.9159 (3)	−0.0475 (3)	0.11218 (16)	0.0431 (8)	
C7	0.8545 (3)	−0.1397 (3)	0.13938 (15)	0.0451 (8)	
H4	0.8874	−0.2124	0.1465	0.054*	
C8	0.7240 (3)	−0.2462 (4)	0.1852 (2)	0.0601 (11)	
H5	0.7502	−0.3157	0.1642	0.072*	
H6	0.7514	−0.2484	0.2260	0.072*	
C9	0.6108 (3)	−0.2557 (3)	0.18749 (19)	0.0594 (11)	
H7	0.5932	−0.3335	0.2039	0.071*	
H8	0.5835	−0.2521	0.1468	0.071*	
C10	0.5605 (3)	−0.1597 (4)	0.22441 (18)	0.0576 (10)	
H9	0.4938	−0.1873	0.2365	0.069*	
H10	0.6010	−0.1451	0.2606	0.069*	
C11	0.4605 (3)	−0.0025 (3)	0.18742 (15)	0.0431 (8)	
H11	0.4089	−0.0455	0.2059	0.052*	
C12	0.4305 (2)	0.1090 (3)	0.15813 (14)	0.0368 (7)	
C13	0.3254 (3)	0.1336 (4)	0.15613 (16)	0.0482 (9)	
H12	0.2801	0.0778	0.1715	0.058*	
C14	0.2889 (3)	0.2375 (4)	0.13210 (18)	0.0530 (9)	
H13	0.2190	0.2515	0.1302	0.064*	
C15	0.3559 (3)	0.3222 (3)	0.11042 (16)	0.0459 (8)	
H14	0.3314	0.3941	0.0946	0.055*	
C16	0.4587 (2)	0.3001 (3)	0.11238 (14)	0.0368 (7)	
C17	0.4991 (2)	0.1913 (3)	0.13444 (13)	0.0322 (6)	
C18	0.5015 (3)	0.5034 (3)	0.0877 (2)	0.0700 (13)	
H15	0.4575	0.5132	0.0532	0.105*	
H16	0.5608	0.5530	0.0835	0.105*	
H17	0.4658	0.5265	0.1233	0.105*	
C19	0.9595 (3)	0.3228 (4)	0.0049 (2)	0.0700 (13)	
H18	1.0106	0.3659	0.0275	0.105*	
H19	0.9145	0.3788	−0.0148	0.105*	
H20	0.9917	0.2736	−0.0247	0.105*	
C20	0.6496 (3)	−0.0934 (4)	0.01313 (18)	0.0659 (12)	
H22	0.7123	−0.0503	0.0081	0.099*	
H23	0.6061	−0.0807	−0.0214	0.099*	
H24	0.6639	−0.1775	0.0173	0.099*	
C21	0.6865 (4)	0.1415 (5)	0.2730 (2)	0.0808 (15)	
H26	0.6287	0.1829	0.2560	0.121*	
H27	0.7315	0.1984	0.2920	0.121*	
H28	0.6638	0.0845	0.3023	0.121*	
C22	0.0864 (6)	0.5031 (8)	0.1513 (3)	0.149 (3)	
H30	0.0580	0.4386	0.1279	0.223*	
H31	0.1406	0.5403	0.1296	0.223*	
H32	0.0344	0.5614	0.1591	0.223*	
C23	0.0337 (5)	0.1674 (7)	0.2229 (3)	0.117 (2)	
H34	0.0975	0.1417	0.2402	0.175*	
H35	0.0392	0.1713	0.1800	0.175*	
H36	−0.0187	0.1113	0.2333	0.175*	
La1	0.716978 (13)	0.298199 (16)	0.077571 (8)	0.03422 (6)	
N1	0.7614 (2)	−0.1353 (2)	0.15504 (12)	0.0418 (6)	
N2	0.5498 (2)	−0.0480 (2)	0.19058 (12)	0.0390 (6)	
N3	0.7794 (3)	0.3947 (3)	0.20112 (16)	0.0617 (9)	
N4	0.7756 (3)	0.5503 (3)	0.03456 (19)	0.0611 (9)	
N5	0.6410 (3)	0.2140 (3)	−0.04698 (17)	0.0621 (9)	
Ni2	0.67227 (3)	0.01383 (3)	0.146167 (17)	0.03239 (10)	
O1	0.78086 (15)	0.09678 (19)	0.09686 (10)	0.0356 (5)	
O2	0.90251 (18)	0.2485 (2)	0.04465 (11)	0.0470 (6)	
O3	0.59889 (15)	0.17423 (19)	0.13315 (10)	0.0356 (5)	
O4	0.53172 (17)	0.3812 (2)	0.09263 (11)	0.0443 (6)	
O5	0.6940 (2)	0.4204 (3)	0.17893 (15)	0.0735 (9)	
O6	0.8089 (3)	0.4327 (4)	0.24951 (17)	0.1125 (15)	
O7	0.8346 (2)	0.3244 (3)	0.17146 (13)	0.0589 (7)	
O8	0.8196 (3)	0.5040 (3)	0.07811 (17)	0.0959 (12)	
O9	0.7975 (3)	0.6493 (3)	0.0170 (2)	0.1064 (14)	
O10	0.7118 (3)	0.4850 (3)	0.00799 (18)	0.0955 (12)	
O11	0.5842 (2)	0.2296 (3)	−0.00488 (15)	0.0778 (9)	
O12	0.6076 (3)	0.1809 (4)	−0.09563 (17)	0.1162 (16)	
O13	0.7331 (3)	0.2290 (4)	−0.03695 (16)	0.0968 (12)	
O14	0.60041 (19)	−0.0515 (2)	0.06531 (11)	0.0509 (6)	
H21	0.5412	−0.0823	0.0666	0.061*	
O15	0.73884 (19)	0.0805 (2)	0.22691 (11)	0.0502 (6)	
H25	0.7842	0.0402	0.2457	0.060*	
O16	0.1250 (4)	0.4577 (4)	0.2063 (2)	0.1259 (17)	
H29	0.1888	0.4494	0.2018	0.151*	
O17	0.0099 (4)	0.2770 (5)	0.2444 (3)	0.147 (2)	
H33	0.0169	0.3201	0.2133	0.177*	

### Atomic displacement parameters (Å^2^)

**Table:**

	*U*^11^	*U*^22^	*U*^33^	*U*^12^	*U*^13^	*U*^23^
C1	0.0312 (15)	0.0446 (18)	0.0340 (16)	0.0024 (14)	−0.0012 (12)	−0.0059 (14)
C2	0.0315 (16)	0.0459 (19)	0.0431 (18)	−0.0004 (15)	0.0033 (14)	−0.0053 (15)
C3	0.0344 (17)	0.067 (3)	0.060 (2)	−0.0023 (18)	0.0070 (16)	−0.012 (2)
C4	0.0324 (18)	0.081 (3)	0.080 (3)	0.011 (2)	0.0056 (19)	−0.008 (2)
C5	0.049 (2)	0.060 (2)	0.067 (3)	0.020 (2)	−0.0034 (19)	−0.006 (2)
C6	0.0384 (17)	0.0447 (19)	0.0462 (19)	0.0099 (15)	−0.0024 (15)	−0.0058 (15)
C7	0.053 (2)	0.0370 (18)	0.0453 (19)	0.0136 (16)	−0.0050 (16)	0.0010 (15)
C8	0.072 (3)	0.0383 (19)	0.070 (3)	0.008 (2)	0.003 (2)	0.0148 (19)
C9	0.079 (3)	0.0354 (18)	0.063 (3)	−0.013 (2)	−0.009 (2)	0.0147 (18)
C10	0.057 (2)	0.057 (2)	0.059 (2)	−0.007 (2)	0.0079 (19)	0.028 (2)
C11	0.0400 (17)	0.050 (2)	0.0393 (18)	−0.0118 (16)	0.0064 (14)	0.0010 (15)
C12	0.0349 (15)	0.0425 (17)	0.0330 (16)	−0.0029 (15)	0.0039 (12)	−0.0029 (14)
C13	0.0346 (17)	0.060 (2)	0.050 (2)	−0.0089 (17)	0.0062 (15)	−0.0038 (18)
C14	0.0301 (17)	0.069 (3)	0.060 (2)	0.0017 (18)	0.0024 (16)	−0.001 (2)
C15	0.0372 (17)	0.052 (2)	0.049 (2)	0.0082 (16)	−0.0002 (15)	−0.0011 (16)
C16	0.0319 (15)	0.0423 (17)	0.0363 (16)	−0.0011 (15)	0.0025 (12)	−0.0015 (14)
C17	0.0310 (14)	0.0381 (16)	0.0277 (14)	−0.0014 (13)	0.0022 (11)	−0.0043 (12)
C18	0.055 (2)	0.035 (2)	0.121 (4)	0.0074 (18)	0.007 (3)	0.003 (2)
C19	0.061 (3)	0.060 (3)	0.090 (3)	−0.007 (2)	0.034 (2)	0.010 (2)
C20	0.070 (3)	0.076 (3)	0.052 (2)	−0.001 (2)	0.003 (2)	−0.021 (2)
C21	0.098 (4)	0.085 (3)	0.060 (3)	0.016 (3)	−0.001 (3)	−0.020 (3)
C22	0.155 (7)	0.177 (8)	0.112 (6)	0.019 (6)	−0.064 (5)	−0.007 (5)
C23	0.092 (5)	0.143 (7)	0.115 (5)	−0.007 (5)	−0.012 (4)	−0.039 (5)
La1	0.03002 (9)	0.03160 (10)	0.04102 (11)	−0.00190 (8)	0.00050 (7)	0.00396 (8)
N1	0.0503 (17)	0.0328 (14)	0.0423 (16)	0.0019 (13)	−0.0010 (13)	0.0016 (12)
N2	0.0446 (15)	0.0386 (14)	0.0339 (14)	−0.0070 (13)	0.0011 (12)	0.0049 (11)
N3	0.067 (2)	0.062 (2)	0.056 (2)	−0.0094 (19)	0.0026 (17)	−0.0179 (18)
N4	0.0447 (18)	0.0439 (18)	0.095 (3)	0.0017 (15)	0.0128 (18)	0.0207 (19)
N5	0.058 (2)	0.068 (2)	0.059 (2)	−0.0034 (19)	−0.0150 (18)	−0.0095 (18)
Ni2	0.0339 (2)	0.03021 (19)	0.0331 (2)	−0.00145 (17)	0.00085 (15)	0.00297 (16)
O1	0.0286 (10)	0.0347 (11)	0.0436 (12)	0.0027 (9)	0.0029 (9)	0.0027 (10)
O2	0.0375 (12)	0.0459 (13)	0.0578 (15)	−0.0013 (11)	0.0123 (11)	0.0073 (12)
O3	0.0284 (10)	0.0345 (11)	0.0439 (12)	0.0007 (9)	0.0029 (9)	0.0058 (9)
O4	0.0358 (12)	0.0323 (12)	0.0648 (16)	0.0040 (10)	0.0035 (11)	0.0042 (11)
O5	0.0490 (16)	0.088 (2)	0.084 (2)	0.0074 (16)	−0.0002 (15)	−0.0301 (18)
O6	0.099 (3)	0.156 (4)	0.081 (3)	−0.001 (3)	−0.018 (2)	−0.066 (3)
O7	0.0612 (17)	0.0586 (17)	0.0564 (16)	0.0114 (14)	−0.0124 (13)	−0.0126 (13)
O8	0.132 (3)	0.067 (2)	0.087 (3)	−0.038 (2)	−0.028 (2)	0.0169 (19)
O9	0.074 (2)	0.056 (2)	0.189 (4)	−0.0085 (18)	0.007 (2)	0.056 (2)
O10	0.088 (2)	0.084 (2)	0.113 (3)	−0.0284 (19)	−0.031 (2)	0.050 (2)
O11	0.0567 (17)	0.114 (3)	0.0626 (18)	−0.0191 (18)	−0.0022 (15)	−0.0078 (18)
O12	0.107 (3)	0.165 (4)	0.076 (3)	−0.007 (3)	−0.033 (2)	−0.049 (3)
O13	0.064 (2)	0.153 (4)	0.073 (2)	−0.011 (2)	0.0007 (17)	−0.035 (2)
O14	0.0452 (13)	0.0647 (16)	0.0429 (14)	−0.0058 (13)	−0.0006 (11)	−0.0085 (12)
O15	0.0513 (14)	0.0556 (15)	0.0436 (13)	0.0066 (13)	−0.0059 (11)	−0.0066 (12)
O16	0.134 (4)	0.129 (4)	0.113 (3)	−0.016 (3)	−0.068 (3)	0.004 (3)
O17	0.117 (4)	0.135 (4)	0.187 (6)	0.001 (3)	−0.069 (4)	0.005 (4)

### Geometric parameters (Å, °)

**Table:**

C1—O1	1.334 (4)	C20—O14	1.417 (4)
C1—C6	1.405 (5)	C20—H22	0.9600
C1—C2	1.405 (5)	C20—H23	0.9600
C2—C3	1.370 (5)	C20—H24	0.9600
C2—O2	1.386 (4)	C21—O15	1.417 (5)
C3—C4	1.378 (6)	C21—H26	0.9600
C3—H1	0.9300	C21—H27	0.9600
C4—C5	1.361 (6)	C21—H28	0.9600
C4—H2	0.9300	C22—O16	1.410 (7)
C5—C6	1.412 (5)	C22—H30	0.9600
C5—H3	0.9300	C22—H31	0.9600
C6—C7	1.445 (5)	C22—H32	0.9600
C7—N1	1.276 (4)	C23—O17	1.349 (6)
C7—H4	0.9300	C23—H34	0.9600
C8—C9	1.491 (6)	C23—H35	0.9600
C8—N1	1.493 (5)	C23—H36	0.9600
C8—H5	0.9700	La1—O3	2.429 (2)
C8—H6	0.9700	La1—O1	2.431 (2)
C9—C10	1.507 (6)	La1—O10	2.594 (3)
C9—H7	0.9700	La1—O7	2.594 (3)
C9—H8	0.9700	La1—O2	2.613 (2)
C10—N2	1.460 (4)	La1—O11	2.623 (3)
C10—H9	0.9700	La1—O4	2.628 (2)
C10—H10	0.9700	La1—O5	2.655 (3)
C11—N2	1.278 (4)	La1—O8	2.659 (3)
C11—C12	1.455 (5)	La1—O13	2.673 (4)
C11—H11	0.9300	La1—Ni2	3.5692 (10)
C12—C17	1.394 (4)	N1—Ni2	2.040 (3)
C12—C13	1.406 (5)	N2—Ni2	2.022 (3)
C13—C14	1.359 (6)	N3—O6	1.214 (4)
C13—H12	0.9300	N3—O5	1.251 (4)
C14—C15	1.383 (5)	N3—O7	1.261 (4)
C14—H13	0.9300	N4—O9	1.207 (4)
C15—C16	1.371 (4)	N4—O8	1.233 (5)
C15—H14	0.9300	N4—O10	1.250 (5)
C16—O4	1.394 (4)	N5—O12	1.218 (4)
C16—C17	1.408 (4)	N5—O11	1.218 (5)
C17—O3	1.324 (3)	N5—O13	1.238 (5)
C18—O4	1.421 (4)	Ni2—O1	2.034 (2)
C18—H15	0.9600	Ni2—O3	2.048 (2)
C18—H16	0.9600	Ni2—O15	2.119 (2)
C18—H17	0.9600	Ni2—O14	2.145 (2)
C19—O2	1.431 (4)	O14—H21	0.8500
C19—H18	0.9600	O15—H25	0.8501
C19—H19	0.9600	O16—H29	0.8500
C19—H20	0.9600	O17—H33	0.8501
			
O1—C1—C6	123.5 (3)	O1—La1—O11	94.47 (10)
O1—C1—C2	118.9 (3)	O10—La1—O11	78.79 (12)
C6—C1—C2	117.6 (3)	O7—La1—O11	167.40 (10)
C3—C2—O2	123.7 (3)	O2—La1—O11	110.72 (10)
C3—C2—C1	122.5 (3)	O3—La1—O4	62.36 (7)
O2—C2—C1	113.8 (3)	O1—La1—O4	128.28 (7)
C2—C3—C4	119.5 (4)	O10—La1—O4	77.19 (10)
C2—C3—H1	120.2	O7—La1—O4	113.45 (9)
C4—C3—H1	120.2	O2—La1—O4	168.09 (7)
C5—C4—C3	120.0 (4)	O11—La1—O4	65.55 (10)
C5—C4—H2	120.0	O3—La1—O5	77.13 (9)
C3—C4—H2	120.0	O1—La1—O5	111.52 (9)
C4—C5—C6	121.8 (4)	O10—La1—O5	95.35 (13)
C4—C5—H3	119.1	O7—La1—O5	48.14 (9)
C6—C5—H3	119.1	O2—La1—O5	117.74 (9)
C1—C6—C5	118.6 (3)	O11—La1—O5	131.30 (10)
C1—C6—C7	124.3 (3)	O4—La1—O5	66.02 (8)
C5—C6—C7	117.1 (3)	O3—La1—O8	144.62 (11)
N1—C7—C6	129.0 (3)	O1—La1—O8	128.46 (11)
N1—C7—H4	115.5	O10—La1—O8	47.10 (11)
C6—C7—H4	115.5	O7—La1—O8	66.71 (10)
C9—C8—N1	114.1 (3)	O2—La1—O8	73.12 (11)
C9—C8—H5	108.7	O11—La1—O8	125.80 (11)
N1—C8—H5	108.7	O4—La1—O8	99.53 (11)
C9—C8—H6	108.7	O5—La1—O8	67.57 (12)
N1—C8—H6	108.7	O3—La1—O13	112.47 (10)
H5—C8—H6	107.6	O1—La1—O13	82.52 (11)
C8—C9—C10	114.4 (4)	O10—La1—O13	70.38 (14)
C8—C9—H7	108.7	O7—La1—O13	138.12 (10)
C10—C9—H7	108.7	O2—La1—O13	65.38 (10)
C8—C9—H8	108.7	O11—La1—O13	46.49 (10)
C10—C9—H8	108.7	O4—La1—O13	108.09 (10)
H7—C9—H8	107.6	O5—La1—O13	165.67 (13)
N2—C10—C9	111.3 (3)	O8—La1—O13	101.83 (13)
N2—C10—H9	109.4	O3—La1—Ni2	33.59 (5)
C9—C10—H9	109.4	O1—La1—Ni2	33.26 (5)
N2—C10—H10	109.4	O10—La1—Ni2	164.67 (8)
C9—C10—H10	109.4	O7—La1—Ni2	81.68 (7)
H9—C10—H10	108.0	O2—La1—Ni2	95.28 (6)
N2—C11—C12	127.2 (3)	O11—La1—Ni2	85.90 (8)
N2—C11—H11	116.4	O4—La1—Ni2	95.69 (5)
C12—C11—H11	116.4	O5—La1—Ni2	94.03 (8)
C17—C12—C13	119.9 (3)	O8—La1—Ni2	148.23 (8)
C17—C12—C11	124.0 (3)	O13—La1—Ni2	99.65 (10)
C13—C12—C11	116.0 (3)	C7—N1—C8	114.4 (3)
C14—C13—C12	121.2 (3)	C7—N1—Ni2	123.7 (2)
C14—C13—H12	119.4	C8—N1—Ni2	121.7 (2)
C12—C13—H12	119.4	C11—N2—C10	116.6 (3)
C13—C14—C15	119.7 (3)	C11—N2—Ni2	125.0 (2)
C13—C14—H13	120.1	C10—N2—Ni2	118.1 (2)
C15—C14—H13	120.1	O6—N3—O5	122.7 (4)
C16—C15—C14	119.8 (3)	O6—N3—O7	120.2 (4)
C16—C15—H14	120.1	O5—N3—O7	117.0 (3)
C14—C15—H14	120.1	O9—N4—O8	121.7 (4)
C15—C16—O4	123.7 (3)	O9—N4—O10	122.7 (4)
C15—C16—C17	122.1 (3)	O8—N4—O10	115.5 (3)
O4—C16—C17	114.3 (3)	O12—N5—O11	120.7 (4)
O3—C17—C12	124.0 (3)	O12—N5—O13	122.5 (4)
O3—C17—C16	118.9 (3)	O11—N5—O13	116.7 (4)
C12—C17—C16	117.1 (3)	N2—Ni2—O1	171.07 (10)
O4—C18—H15	109.5	N2—Ni2—N1	97.73 (12)
O4—C18—H16	109.5	O1—Ni2—N1	90.95 (10)
H15—C18—H16	109.5	N2—Ni2—O3	89.47 (10)
O4—C18—H17	109.5	O1—Ni2—O3	81.91 (8)
H15—C18—H17	109.5	N1—Ni2—O3	172.70 (10)
H16—C18—H17	109.5	N2—Ni2—O15	91.47 (10)
O2—C19—H18	109.5	O1—Ni2—O15	90.85 (10)
O2—C19—H19	109.5	N1—Ni2—O15	88.59 (11)
H18—C19—H19	109.5	O3—Ni2—O15	90.02 (10)
O2—C19—H20	109.5	N2—Ni2—O14	87.22 (10)
H18—C19—H20	109.5	O1—Ni2—O14	90.25 (9)
H19—C19—H20	109.5	N1—Ni2—O14	92.79 (11)
O14—C20—H22	109.5	O3—Ni2—O14	88.74 (10)
O14—C20—H23	109.5	O15—Ni2—O14	178.21 (10)
H22—C20—H23	109.5	N2—Ni2—La1	130.47 (8)
O14—C20—H24	109.5	O1—Ni2—La1	40.96 (6)
H22—C20—H24	109.5	N1—Ni2—La1	131.79 (8)
H23—C20—H24	109.5	O3—Ni2—La1	41.00 (6)
O15—C21—H26	109.5	O15—Ni2—La1	89.01 (7)
O15—C21—H27	109.5	O14—Ni2—La1	90.90 (8)
H26—C21—H27	109.5	C1—O1—Ni2	126.7 (2)
O15—C21—H28	109.5	C1—O1—La1	124.71 (19)
H26—C21—H28	109.5	Ni2—O1—La1	105.78 (8)
H27—C21—H28	109.5	C2—O2—C19	117.9 (3)
O16—C22—H30	109.5	C2—O2—La1	118.70 (19)
O16—C22—H31	109.5	C19—O2—La1	123.3 (2)
H30—C22—H31	109.5	C17—O3—Ni2	125.81 (19)
O16—C22—H32	109.5	C17—O3—La1	124.66 (18)
H30—C22—H32	109.5	Ni2—O3—La1	105.41 (9)
H31—C22—H32	109.5	C16—O4—C18	116.9 (3)
O17—C23—H34	109.5	C16—O4—La1	117.03 (18)
O17—C23—H35	109.5	C18—O4—La1	125.8 (2)
H34—C23—H35	109.5	N3—O5—La1	96.0 (2)
O17—C23—H36	109.5	N3—O7—La1	98.7 (2)
H34—C23—H36	109.5	N4—O8—La1	97.2 (2)
H35—C23—H36	109.5	N4—O10—La1	100.0 (2)
O3—La1—O1	66.81 (7)	N5—O11—La1	99.9 (2)
O3—La1—O10	138.52 (10)	N5—O13—La1	96.8 (3)
O1—La1—O10	148.37 (11)	C20—O14—Ni2	126.8 (2)
O3—La1—O7	91.75 (9)	C20—O14—H21	108.8
O1—La1—O7	76.26 (8)	Ni2—O14—H21	119.9
O10—La1—O7	113.57 (11)	C21—O15—Ni2	125.7 (3)
O3—La1—O2	128.84 (7)	C21—O15—H25	104.0
O1—La1—O2	62.21 (7)	Ni2—O15—H25	120.5
O10—La1—O2	91.05 (11)	C22—O16—H29	106.2
O7—La1—O2	72.79 (9)	C23—O17—H33	101.2
O3—La1—O11	76.60 (9)		

### Hydrogen-bond geometry (Å, °)

**Table:**

*D*—H···*A*	*D*—H	H···*A*	*D*···*A*	*D*—H···*A*
O17—H33···O16	0.85	2.10	2.665 (7)	124
O15—H25···O16^i^	0.85	1.83	2.681 (5)	174
O14—H21···O12^ii^	0.85	2.34	3.169 (5)	165
O16—H29···O15^iii^	0.85	2.35	2.681 (5)	104

Symmetry codes: (i) −*x*+1, *y*−1/2, −*z*+1/2; (ii) −*x*+1, −*y*, −*z*; (iii) −*x*+1, *y*+1/2, −*z*+1/2.
